# Supplementary material for: Altered Functional Connectivity of Basal Ganglia in Mild Cognitive Impairment and Alzheimer’s Disease
Source: Brain Sci. 2022 Nov 15;12(11):1555. doi: 10.3390/brainsci12111555 (PMC9688931; doi:10.3390/brainsci12111555)
Supplement: Supplementary file 1 [file brainsci-12-01555-s001.zip › brainsci-2012549-supplementary.pdf]

Table S1.FCD among-group comparison in the AD,MCI and NC groups (voxel-level $p < 0.05$, cluster-level $p < 0.05$, GRF corrected)

Conditions	Region	Brodmann's Area	Montreal Neurologic Institute Coordinates	T value	Cluster (Voxels)
MCI VS NC					
Short-range	Caudate_R	-	(15,-9,19)	2.6643	35
	Caudate_L	-	(-19,-17,22)	2.0899	7
	Putamen_R	BA48	(33,-10,-1)	2.9346	32
	Putamen_L	BA48	(-29,-13,6)	2.4678	19
	Amygdala_R	BA34	(22,-3,-17)	2.9021	24
	Pallidum_R	-	(29,-9,-3)	2.2932	14
AD VS NC					
Long-range	Caudate_L	-	(-7,6,11)	-3.2014	67
	Pallidum_R	BA48	(21,-3,-3)	-2.3434	8

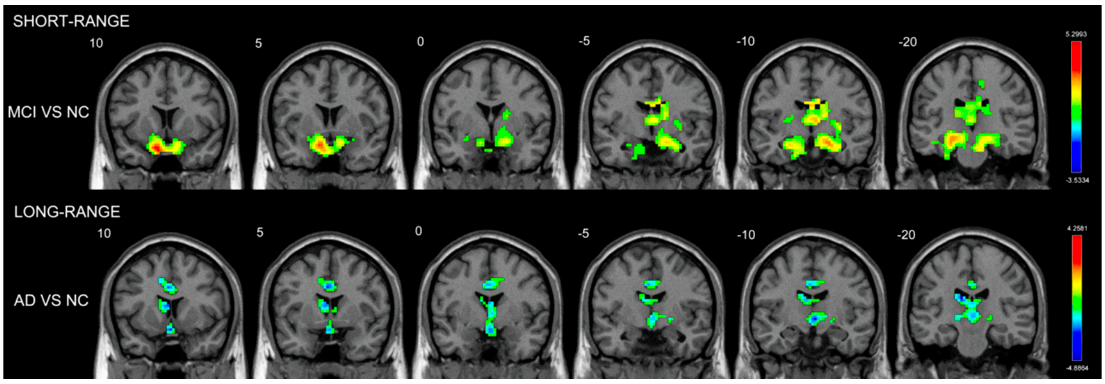

Figure S1. Brain regions with significant differences in FCD (voxel-level $p < 0.05$, cluster-level $p < 0.05$, GRF corrected) among the AD, MCI and NC groups.

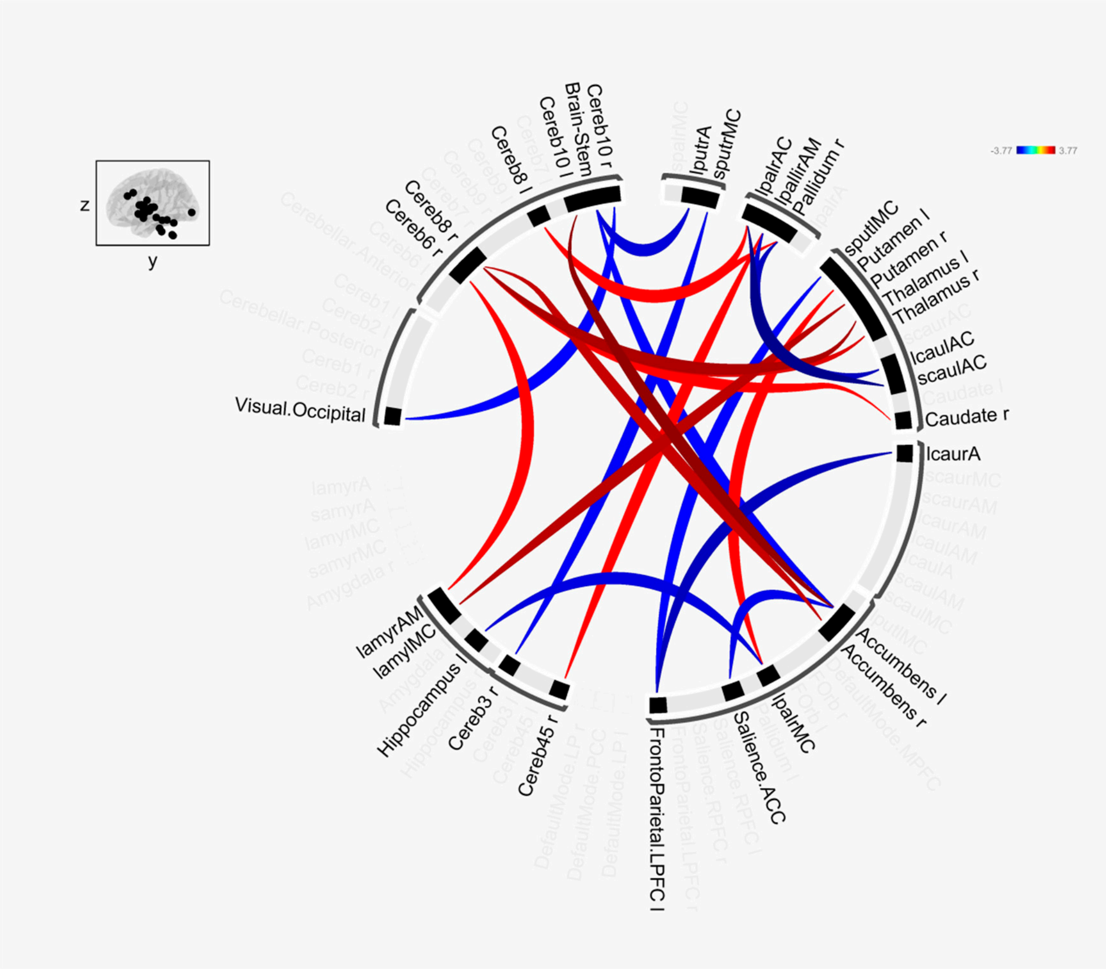

Figure S2. Functional connectivity with significant differences (voxel-level $p < 0.01$, cluster-level $p < 0.05$, no correction) between the MCI patients and normal controls.
